# Association between dietary carotenoids intake and chronic constipation in American men and women adults: a cross-sectional study

**DOI:** 10.1186/s12889-023-16367-3

**Published:** 2023-08-22

**Authors:** Jiangnan Wang, Wanru Kong, Min Liu, Yuping Wang, Ya Zheng, Yongning Zhou

**Affiliations:** 1 The First Clinical Medical College, Lanzhou University, Lanzhou, 730000 China; 2 Department of Gastroenterology, The First Hospital of Lanzhou University, Lanzhou, 730000 China; 3 Key Laboratory for Gastrointestinal Diseases of Gansu Province, Department of Gastroenterology, The First Hospital of Lanzhou University, Lanzhou, 730000 China; 4 Department of Infection Management, Gansu Provincial Hospital, Lanzhou, 730000 China

**Keywords:** Carotenoids, Chronic constipation, Lycopene, α-Carotene, NHANES

## Abstract

**Background:**

Dietary carotenoids have been proven to improve intestinal disorders like inflammatory bowel disease and colon cancer, yet little is known about the link between dietary carotenoids and constipation. This study aims to examine the relationship between dietary carotenoids intake and constipation, using data from the National Health and Nutrition Examination Survey (NHANES) 2005–2010.

**Methods:**

A total of 11,722 participants were enrolled. Chronic constipation was defined as type 1 (separate hard lumps, like nuts) and type 2 (sausage-like, but lumpy) in the Bristol stool form scale (BSFS). Carotenoids intake was obtained from the average of two 24-hour dietary recall questionnaires (if only one 24-hour was available, we used it) and divided into quartiles (Q). The prevalence of constipation was calculated across men and women individuals. The relationship between dietary carotenoids intake and constipation in men and women was assessed with weighted logistic regression and smoothed curve fitting after adjusting confounders, with results displayed as weighted odds ratio (OR) with 95% confidence intervals (95% CI). The model was further stratified by age, race, and HEI 2015 scores (with median as cutoff) among men and women.

**Results:**

The total weighted prevalence of chronic constipation in this study was 8.08%, 11.11% in women and 5.18% in men. After multivariable adjustment, compared with the lowest intake, participants with the highest dietary lycopene intake (OR_Q4 vs. Q1=_ 0.55, 95% CI: 0.36–0.84, p for trend = 0.01) and total lycopene intake (OR_Q4 vs. Q1_ = 0.52, 95% CI: 0.34–0.80, p for trend = 0.01) were negatively associated with the risk of chronic constipation in men, whereas increased dietary α-carotene intake reduced the risk of chronic constipation in women (OR_Q4 vs. Q1_ = 0.69, 95% CI: 0.48–0.98, p for trend = 0.04). Smoothing curve fitting further supported these results and provided evidence of dose-response effects. No association was found between other types of carotenoids and chronic constipation in men and women.

**Conclusions:**

Increasing lycopene intake may improve bowel function in men while increased α-carotene intake may reduce the risk of chronic constipation in women. Further studies are essential to explore the role that the intake of carotenoids plays in chronic constipation.

## Background

Patients with chronic constipation suffer a significant burden of disease and suffer from low quality of life. According to estimates, chronic constipation affects between 10.1% and 15.3% of the adult population worldwide, with a higher prevalence observed among women compared to men [1], and its prevalence increases with age [2]. Chronic constipation is a multifactorial disease, with common risk factors such as genetic predisposition, socio-economic status, parental education, lifestyle, drugs and depression [2, 3]. Lifestyle is a modifiable risk factor for chronic constipation; in particular, the relationship between dietary components and chronic constipation has attracted much attention. Recent studies have reported that soluble fiber and microelements, such as selenium, magnesium and phosphorus have been recognized to decrease chronic constipation risk [3–6], while high dietary saturated fat or women’s relatively low energy intake is linked to increased risk [7, 8]. Therefore, the investigation of potential dietary strategies for chronic constipation holds promise for both the prevention and management of the disease.

Carotenoids, a group of natural pigments that cannot be produced by the human body and only be obtained through dietary sources, are widely found in fruits and vegetables with numerous biological activities such as antioxidant, anti-inflammatory, regulating cell growth, regulating immune function, and anti-cancer [9, 10]. Previous research findings have suggested that carotenoids intake may reduce the risk of intestinal diseases such as colon cancer and improve ulcerative colitis [11–13]. Furthermore, patients in remission from ulcerative colitis who consumed higher lutein and zeaxanthin reported less constipation [14]. Moreover, traditional plants that could treat constipation are rich in carotenoids, for example, Rosa spp. contains lycopene, and β-carotene is one of the main components of Aloe in treating constipation [15, 16]. Nevertheless, limited research has been conducted to investigate the impact of carotenoids on chronic constipation.

Based on NHANES data, we (1) calculated the prevalence of chronic constipation among American adults, (2) measured the association between dietary carotenoids consumption and chronic constipation, and (3) investigated the potential influence of age, gender, race and diet quality with respect to the relationship between carotenoids intake and chronic constipation.

## Methods

### Data source

The National Health and Nutrition Examination Survey (NHANES) is a national sample survey conducted by the National Center for Health Statistics (NCHS), aims to measure the health and nutritional status of adults and children in the non-institutionalized civilian population of the United States [4, 5, 8].

### Study population

**Fig. 1:**
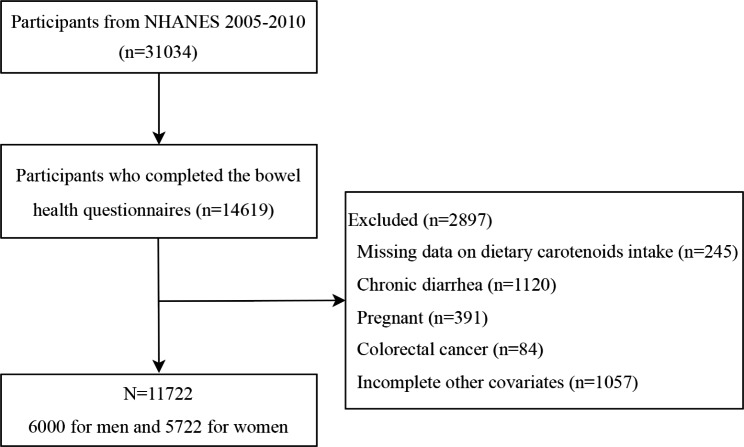
Flow chart of participants selection

The data used in this study came from three consecutive cycles of the NHANES, which covered the years 2005–2006, 2007–2008, and 2009–2010, as they contained both carotenoids diet data and bowel health questionnaires (BHQ) data, with a total of 31,034 participants in the three cycles, of which 14,619 were included due to complete BHQ information. An additional 2897 participants were excluded because they did not fulfill the criteria for inclusion: (1) missing information on dietary carotenoids intake (n = 245); (2) self-reported chronic diarrhea (n = 1120); (3) pregnancy (n = 391); (4) self-reported colorectal cancer (n = 84); or (5) incomplete information on other variables (n = 1057). Finally, 11,722 participants were included in the final analysis (flowchart shown in Fig. 1). A total of 947 participants were diagnosed with chronic constipation, 311 of whom were men and 636 were women.

### Diagnosis of constipation

Based on previous studies, chronic constipation could be assessed by the Bristol Stool Form Scale (BSFS), which was provided by the Mobile Examination Center (MEC) interview and administered to adults aged 20 years and older. Participants were told to look at a card and identify their usual or most frequent stool type by the number on it. Chronic constipation was composed of type 1 (separate hard lumps, like nuts) and type 2 (sausage-like, but lumpy) in BSFS; type 6 (fluffy pieces with ragged edges, a mushy stool) and type 7 (watery, no solid pieces) were classified as diarrhea; and type 3 (like a sausage but with cracks in the surface), type 4 (like a sausage or snake, smooth and soft) and type 5 (soft blobs with clear-cut edges) were considered to have a healthy bowel [4, 8].

### Measures of dietary carotenoids

Two 24-hour dietary recall questionnaires were administered by NHANES, the first by face-to-face collection at MEC, and the second by telephone collection 3 to 10 days later. This provided dietary intakes of α-carotene, β-carotene, β-cryptoxanthin, lycopene and lutein with zeaxanthin (combined). The consumption of lycopene, as well as lutein with zeaxanthin supplements over the same two 24-hour periods was also recorded (from 2007 onwards). Intake of carotenoids was calculated using the average of two 24-hour recalls or the reported value for participants who completed one 24-hour interview. Intake of total lycopene and total lutein with zeaxanthin intake were determined from dietary and supplemental sources.

### Covariates

We collected covariates associated with constipation and carotenoids intake through literature review. These variables included age, race (Mexican American, non-Hispanic black, non-Hispanic white, and other races), marital status (living alone, married or living with a partner), educational levels (below high school, high school, above high school), poverty-income ratio (PIR, < 2 or ≥ 2), body mass index (BMI), smoking, drinking, vigorous physical activity, hypertension, diabetes, depression, HEI 2015 scores and dietary intake, including energy, fat, fiber, and fluid. BMI was grouped as underweight or normal (< 25 kg/m^2^), overweight (25–30 kg/m^2^), and obese (> 30 kg/m^2^). Classification of smoking was as follows: Never: smoked under 100 cigarettes in their lifetime; Former: smoked over 100 cigarettes in their lifetime and smoke not at all now; and Now: smoked over 100 cigarettes in their lifetime and smoke some or all of the time. Drinking was defined if a person has had at least 12 drinks a year. The definition of vigorous physical activity varied by year. In the cycle of 2005–2006, it was defined as doing any vigorous activities for at least 10 minutes that caused heavy sweating, or significant elevations in breathing or heart rate over the past 30 days. While during 2007–2010, those who answered yes to the question ‘Work involve vigorous-intensity activities that cause significant increases in breathing or heart rate, such as carrying or lifting heavy loads, digging, or construction work for at least 10 minutes continuously’ were considered to have vigorous physical activity. Those who met any of the following criteria were considered hypertensive: (1) informed by a medical professional; (2) used anti-hypertensive medication; or (3) average systolic blood pressure of at least 130 mmHg or average diastolic blood pressure of at least 80 mmHg. The diagnostic criteria for diabetes were: (1) doctor told; or (2) glycohemoglobin HbA1c ≥ 6.5%; or (3) use of diabetes medication or insulin. Depression information from the PHQ9 questionnaire, with scores ≥ 10, was considered positive. The intake of dietary nutrients and HEI 2015 scores were obtained from two 24-hour dietary interviews (the reported value was used if it was available only).

### Statistical analysis

Taking into consideration NHANES’ complex sample design, we weighted the data with appropriate weights based on NHANES guidelines (one-third of the 2005–2010 weight). Continuous variables were displayed as weighted means (SE) and comparisons between groups were tested using t-tests, and survey-weighted percentages (SE) were used to describe categorical variables, while chi-square tests were performed on comparing groups.

An analysis of multivariable logistic regression was conducted to analyze the relationship between dietary carotenoids intake and chronic constipation among men and women, with results shown as weighted Odds Ratio (OR) with 95% confidence intervals (95% CI). Model 1 was not adjusted for any of the variables, while model 2 was adjusted for potential confounding including age (as a continuous variable), race, marital status, educational levels and PIR (as a continuous variable). In model 3, we further adjusted for vigorous physical activity, BMI (as a continuous variable), smoking, drinking, diabetes, hypertension, depression, and dietary energy, dietary fiber, fat, fluid intake, and HEI 2015 scores. To better visualize the association between dietary carotenoids subclass intake and constipation risk, we performed weighted smoothed curve fitting using the generalized additive model (GAM) after fully adjusting for covariates. In addition, models were stratified by age, race and HEI 2015 scores (with median as cutoff) to explore the heterogeneity of results. All data processing was performed in R version 4.2.2 (https://www.r-project.org/). A two-sided *P* < 0.05 was considered statistically significant.

## Results

### Participants’ characteristics

Table 1 shows the participants’ baseline characteristics. According to the diagnostic criteria, 947 of the 11,722 participants screened positive for chronic constipation, 311 (5.18%) in men and 636 (11.11%) in women. Compared with participants who had healthy intestines, men with constipation were often Mexican American, having lower education levels and PIR, having lower BMI, no drinking, and were more likely to be accompanied by depression, having lower dietary intake (energy, fiber, fat, fluid) and unhealthy eating habits (lower HEI 2015 scores). Women with constipation were more likely to be non-Hispanic Black, have lower education levels and PIR, have lower BMI, were non-drinker, have lower fiber, fat, fluid intake and lower HEI 2015 scores and depression in the univariate analysis.

**Table 1:** Characteristics of participants by constipation among men and women, weighted

Characteristic	Males			*p* value	Females			*p* value
	No constipation	Constipation			No constipation	Constipation		
	(n=5689)	(n=311)			(n=5086)	(n=636)		
Age (years), % (SE)				0.12				0.14
20-50	60.46 (1.27)	66.18 (3.47)			56.35 (1.22)	60.34 (2.28)		
≥50	39.54 (1.27)	33.82 (3.47)			43.65 (1.22)	39.66 (2.28)		
Race, % (SE)				< 0.0001				0.03
Mexican American	8.62 (1.00)	16.90 (3.02)			6.67 (0.84)	7.52 (1.24)		
Non-Hispanic Black	10.14 (0.88)	17.80 (2.95)			10.82 (1.05)	14.85 (2.39)		
Non-Hispanic White	71.82 (1.76)	56.10 (4.72)			73.64 (1.82)	67.02 (3.61)		
Other races	9.42 (0.87)	9.21 (2.84)			8.87 (0.82)	10.61 (2.00)		
Marital status, % (SE)				0.06				0.43
Living alone	33.36 (1.13)	42.86 (4.74)			39.04 (1.38)	41.46 (2.76)		
Married or living with partner	66.64 (1.13)	57.14 (4.74)			60.96 (1.38)	58.54 (2.76)		
Educational levels, % (SE)				< 0.0001				< 0.001
Below high school	5.32 (0.45)	10.22 (2.26)			4.09 (0.35)	7.07 (1.17)		
High school	35.61 (1.34)	51.71 (5.28)			35.60 (1.13)	42.48 (2.67)		
Above high school	59.07 (1.48)	38.07 (5.15)			60.31 (1.24)	50.45 (2.92)		
Poverty-income ratio, % (SE)				< 0.001				0.001
< 2	29.99 (1.15)	42.94 (3.99)			33.61 (1.26)	42.37 (2.60)		
≥ 2	70.01 (1.15)	57.06 (3.99)			66.39 (1.26)	57.63 (2.60)		
BMI (kg/m^2^), % (SE)				0.02				0.02
< 25	26.19 (1.17)	37.92 (5.15)			36.30 (1.09)	41.13 (2.67)		
25-30	39.23 (0.94)	35.67 (3.83)			27.99 (1.20)	31.87 (2.44)		
> 30	34.58 (1.19)	26.41 (3.15)			35.71 (1.12)	27.00 (2.30)		
Vigorous physical activity, % (SE)				0.05				0.29
No	64.77 (0.93)	73.53 (3.84)			80.25 (0.85)	82.91 (2.32)		
Yes	35.23 (0.93)	26.47 (3.84)			19.75 (0.85)	17.09 (2.32)		
Smoking status, % (SE)				0.61				0.21
Former	28.14 (1.05)	24.03 (4.08)			21.12 (0.94)	18.69 (1.90)		
Never	46.85 (1.21)	48.71 (4.51)			58.47 (1.12)	62.86 (2.42)		
Now	25.00 (0.88)	27.26 (4.28)			20.41 (0.93)	18.46 (1.95)		
Drinking status, % (SE)				0.01				0.01
No	13.63 (0.82)	21.81 (3.31)			30.73 (1.37)	38.23 (2.82)		
Yes	86.37 (0.82)	78.19 (3.31)			69.27 (1.37)	61.77 (2.82)		
Diabetes, % (SE)				0.12				0.94
No	89.85 (0.43)	92.62 (1.48)			90.46 (0.57)	90.36 (1.17)		
Yes	10.15 (0.43)	7.38 (1.48)			9.54 (0.57)	9.64 (1.17)		
Hypertension, % (SE)				0.43				0.38
No	48.57 (1.20)	51.47 (3.56)			55.37 (1.01)	57.76 (2.63)		
Yes	51.43 (1.20)	48.53 (3.56)			44.63 (1.01)	42.24 (2.63)		
Depression, % (SE)				< 0.001				0.01
No	95.25 (0.46)	83.85 (5.05)			91.87 (0.60)	87.37 (1.55)		
Yes	4.75 (0.46)	16.15 (5.05)			8.13 (0.60)	12.63 (1.55)		
Dietary measures, mean (SE)								
Energy (kcal/day)	2516.61 (21.45)	2313.84 (70.51)		0.01	1772.35 (11.84)	1724.37 (28.64)		0.12
Fiber (gm/day)	18.23 (0.27)	15.32 (0.75)		< 0.001	15.05 (0.19)	13.46 (0.41)		< 0.001
Fat (gm/day)	95.24 (1.11)	83.87 (3.02)		0.001	67.51 (0.66)	63.84 (1.41)		0.02
Fluid (gm/day)	3297.27 (37.22)	2803.36 (103.25)		< 0.0001	2686.75 (22.29)	2408.44 (64.46)		< 0.0001
HEI 2015 scores	51.50 (0.35)	48.59 (0.99)		0.01	54.66 (0.35)	52.22 (0.82)		0.001
Dietary carotenoids intake (mcg/day), mean (SE)								
ɑ-Carotene	403.30 (17.47)	308.17 (46.55)		0.05	427.75 (17.59)	321.99 (33.27)		0.002
β-Carotene	2078.85 (66.34)	1615.60 (191.21)		0.02	2294.29 (69.93)	1751.85 (155.71)		< 0.001
β-Cryptoxanthin	104.68 (4.25)	80.09 (9.84)		0.02	93.72 (4.20)	74.44 (4.76)		< 0.001
Lycopene (Diet)	6709.98 (196.94)	4473.85 (466.80)		< 0.001	4910.82 (177.90)	4528.77 (293.77)		0.28
Lutein with zeaxanthin (Diet)	1431.17 (53.55)	1252.91 (204.11)		0.39	1573.87 (50.04)	1245.21 (97.07)		0.002
Total lycopene (Diet and Supplement)	6798.84 (203.78)	4515.88 (470.57)		< 0.001	4956.40 (178.36)	4553.95 (293.14)		0.25
Total lutein with zeaxanthin (Diet and Supplement)	1496.46 (50.49)	1280.90 (204.72)		0.30	1686.20 (49.59)	1325.74 (98.66)		0.001

*SE* standard errors, *BMI* body mass index, *HEI* healthy eating index

### Association between carotenoids intake and chronic constipation among men and women

The weighted OR (95% CI) between quartiles of carotenoids intake and chronic constipation are shown in Table 2. Among the male participants in the highest quartile of carotenoids intake, the results of logistic regression showed that all carotenoids, with the exception of α-carotene, exhibited an inverse relationship with constipation in model 1 (OR_Q4 vs. Q1_ (95% CI): β-carotene, 0.46 (0.29–0.73); β-cryptoxanthin, 0.64 (0.43–0.96); lycopene, 0.41 (0.25–0.66); lutein with zeaxanthin, 0.46 (0.28–0.75); total lycopene, 0.39 (0.24–0.62); total lutein with zeaxanthin, 0.42 (0.26–0.69)). However, there was no statistically significant association between dietary β-cryptoxanthin intake and chronic constipation in model 2 (OR_Q4 vs. Q1_ = 0.70, 95% CI: 0.48–1.01). After further adjustment for other variables in Model 3, the odds of constipation were decreased across quartiles of dietary lycopene intake (OR_Q4 vs. Q1_ = 0.55, 95% CI: 0.36–0.84, p for trend = 0.01) and total lycopene intake (OR_Q4 vs. Q1_ = 0.52, 95% CI: 0.34–0.80, p for trend = 0.01).

**Table 2:** Weighted ORs and 95% CIs for constipation among men and women according to the quartiles of dietary carotenoids intake

Carotenoids (mcg/day)	Quartile 1	Quartile 2	Quartile 3	Quartile 4	p for trend	
**Men**						
ɑ-Carotene						
Median (Range)	8.5 [0-24]	43 (24-75.5]	170 (75.5-435.375]	1012.5 (435.375-19,519]		
Model 1	Ref.	0.71 (0.48, 1.06)	0.69 (0.44, 1.06)	0.71 (0.48, 1.07)	0.09	
Model 2	Ref.	0.72 (0.48, 1.10)	0.80 (0.52, 1.21)	0.88 (0.58, 1.34)	0.57	
Model 3	Ref.	0.90 (0.60, 1.35)	1.08 (0.66, 1.78)	1.20 (0.74, 1.96)	0.38	
β-Carotene						
Median (Range)	232.5 [0-406]	623.5 (406-970]	1545 (970-2515.625]	4368.5 (2515.625-41,975]		
Model 1	Ref.	**0.60 (0.40, 0.91)**	**0.57 (0.39, 0.82)**	**0.46 (0.29, 0.73)**	**0.001**	
Model 2	Ref.	0.65 (0.42, 0.99)	0.72 (0.50, 1.05)	**0.61 (0.40, 0.93)**	**0.03**	
Model 3	Ref.	0.84 (0.57, 1.24)	0.99 (0.61, 1.60)	0.93 (0.58, 1.48)	0.91	
β-Cryptoxanthin						
Median (Range)	5 [0-12.5]	23 (12.5-41.5]	70.5 (41.5-117.625]	222.75 (117.625-4011]		
Model 1	Ref.	**0.59 (0.42, 0.84)**	0.70 (0.44, 1.13)	**0.64 (0.43, 0.96)**	0.10	
Model 2	Ref.	**0.61 (0.44, 0.86)**	0.76 (0.48, 1.22)	0.70 (0.48, 1.01)	0.18	
Model 3	Ref.	**0.69 (0.50, 0.96)**	0.97 (0.65, 1.45)	0.91 (0.62, 1.34)	0.91	
Lycopene (Diet)						
Median (Range)	6 [0-835.5]	1783.5 (835.5-3110.5]	4965.25 (3110.5-7728.875]	13,459.75 (7728.875-143,241.5]		
Model 1	Ref.	0.87 (0.62, 1.22)	0.69 (0.46, 1.04)	**0.41 (0.25, 0.66)**	**<0.001**	
Model 2	Ref.	0.90 (0.65, 1.26)	0.69 (0.44, 1.09)	**0.44 (0.27, 0.71)**	**0.003**	
Model 3	Ref.	1.02 (0.74, 1.41)	0.86 (0.60, 1.24)	**0.55 (0.36, 0.84)**	**0.01**	
Lutein with zeaxanthin (Diet)						
Median (Range)	261.5 [0-412.5]	573 (412.5-760.25]	1004.5 (760.25-1384.375]	2366.25 (1384.375-47,066]		
Model 1	ref	**0.56 (0.32, 0.96)**	**0.37 (0.21, 0.65)**	**0.46 (0.28, 0.75)**	**0.001**	
Model 2	ref	0.59 (0.35, 1.00)	**0.42 (0.24, 0.73)**	**0.60 (0.38, 0.94)**	**0.01**	
Model 3	Ref.	0.75 (0.45, 1.23)	**0.58 (0.34, 0.96)**	0.93 (0.59, 1.47)	0.39	
Total lycopene (Diet and Supplement)						
Median (Range)	72 [0-886.375]	1863.5 (886.375-3158.75]	5040.75 (3158.75-7823]	13,563 (7823-143,241.5]		
Model 1	Ref.	0.85 (0.58, 1.23)	0.70 (0.47, 1.03)	**0.39 (0.24, 0.62)**	**<0.001**	
Model 2	Ref.	0.88 (0.61, 1.26)	0.70 (0.45, 1.08)	**0.41 (0.25, 0.67)**	**0.001**	
Model 3	Ref.	0.99 (0.70, 1.42)	0.86 (0.61, 1.22)	**0.52 (0.34, 0.80)**	**0.01**	
Total lutein with zeaxanthin (Diet and Supplement)						
Median (Range)	268.5 [0-435.5]	600.5 (435.5-798]	1052.5 (798-1448.5]	2489 (1448.5-47,066]		
Model 1	Ref.	**0.45 (0.27, 0.76)**	**0.44 (0.27, 0.72)**	**0.42 (0.26, 0.69)**	**0.002**	
Model 2	Ref.	**0.48 (0.29, 0.80)**	**0.51 (0.31, 0.82)**	**0.56 (0.35, 0.89)**	**0.02**	
Model 3	Ref.	0.60 (0.36, 0.99)	0.69 (0.45, 1.08)	0.88 (0.55, 1.41)	0.49	
**Women**						
ɑ-Carotene						
Median (Range)	10.5 [0-25.5]	47 (25.5-90.25]	203 (90.25-453]	966 (453-18,675]		
Model 1	Ref.	**0.60 (0.46, 0.78)**	**0.58 (0.43, 0.79)**	**0.53 (0.39, 0.72)**	**<0.0001**	
Model 2	Ref.	**0.63 (0.48, 0.83)**	**0.63 (0.46, 0.88)**	**0.60 (0.43, 0.83)**	**0.002**	
Model 3	Ref.	**0.67 (0.50, 0.91)**	0.70 (0.49, 0.99)	**0.69 (0.48, 0.98)**	**0.04**	
β-Carotene						
Median (Range)	231 [0-416.5]	675 (416.5-1066.5]	1745 (1066.5-2763.75]	4648.5 (2763.75-48,098.5]		
Model 1	Ref.	0.81 (0.59, 1.11)	**0.67 (0.53, 0.86)**	**0.49 (0.37, 0.66)**	**<0.0001**	
Model 2	Ref.	0.88 (0.64, 1.22)	**0.76 (0.58, 0.99)**	**0.57 (0.41, 0.80)**	**<0.001**	
Model 3	Ref.	0.97 (0.68, 1.39)	0.88 (0.63, 1.24)	0.70 (0.47, 1.05)	0.07	
β-Cryptoxanthin						
Median (Range)	5 [0-12]	23 (12-40]	69 (40-110]	195.5 (110-4958]		
Model 1	Ref.	**0.67 (0.48, 0.92)**	**0.71 (0.51, 0.98)**	**0.68 (0.50, 0.92)**	**0.01**	
Model 2	Ref.	**0.69 (0.51, 0.94)**	0.76 (0.56, 1.02)	0.75 (0.54, 1.04)	0.07	
Model 3	Ref.	0.70 (0.49, 1.00)	0.82 (0.59, 1.13)	0.83 (0.56, 1.23)	0.40	
Lycopene(Diet)						
Median (Range)	3 [0-548.25]	1248.75 (548.25-2103]	3512.75 (2103-5840.5]	10,378 (5840.5-126,269]		
Model 1	Ref.	1.01 (0.78, 1.30)	0.87 (0.63, 1.21)	0.91 (0.68, 1.23)	0.41	
Model 2	Ref.	1.07 (0.83, 1.38)	0.94 (0.67, 1.33)	1.00 (0.74, 1.35)	0.80	
Model 3	Ref.	1.14 (0.88, 1.48)	1.02 (0.71, 1.46)	1.16 (0.82, 1.64)	0.56	
Lutein with zeaxanthin (Diet)						
Median (Range)	245.5 [0-396]	545 (396-723.75]	984.75 (723.75-1427.75]	2564.5 (1427.75-57,188]		
Model 1	Ref.	**0.65 (0.47, 0.90)**	**0.67 (0.50, 0.89)**	**0.59 (0.44, 0.80)**	**0.002**	
Model 2	Ref.	**0.69 (0.49, 0.99)**	0.75 (0.56, 0.99)	**0.69 (0.51, 0.94)**	**0.03**	
Model 3	Ref.	0.73 (0.51, 1.06)	0.89 (0.60, 1.31)	0.90 (0.63, 1.28)	0.76	
Total lycopene (Diet and Supplement)						
Median (Range)	17.5 [0-579.625]	1255.5 (579.625-2156]	3553.5 (2156-5896.25]	10,422 (5896.25-126,269]		
Model 1	Ref.	0.91 (0.68, 1.21)	0.84 (0.61, 1.15)	0.85 (0.63, 1.14)	0.26	
Model 2	Ref.	0.97 (0.73, 1.28)	0.91 (0.65, 1.28)	0.93 (0.69, 1.26)	0.59	
Model 3	Ref.	1.01 (0.76, 1.35)	0.97 (0.69, 1.38)	1.06 (0.76, 1.48)	0.79	
Total lutein with zeaxanthin (Diet and Supplement)						
Median (Range)	255 [0-412]	568 (412-757]	1036 (757-1503.25]	2790 (1503.25-57,188]		
Model 1	Ref.	**0.65 (0.49, 0.86)**	**0.67 (0.49, 0.91)**	**0.56 (0.41, 0.76)**	**0.002**	
Model 2	Ref.	**0.70 (0.52, 0.94)**	0.75 (0.55, 1.02)	**0.66 (0.48, 0.89)**	**0.02**	
Model 3	Ref.	0.73 (0.53, 1.01)	0.88 (0.59, 1.32)	0.84 (0.58, 1.20)	0.51	

*OR* odds ratio, *CI* confidence interval

Model 1 did not adjust for any variables

Model 2 adjusted for age (as a continuous variable), race, marital status, educational levels and PIR (as a continuous variable)

Model 3 further adjusted for vigorous physical activity, BMI (as a continuous variable), smoking status, drinking status, diabetes, hypertension, depression, and dietary energy, dietary fiber, fat, fluid intake, and HEI 2015 scores

The bold values refer to P< 0.05

In women, higher dietary intakes of α-carotene, β-carotene, lutein with zeaxanthin and total lutein with zeaxanthin in the fourth quartile (compared to the first quartile) were associated with a reduced risk of chronic constipation. This was observed in both model 1 (OR_Q4 vs. Q1_ (95% CI), α-carotene, 0.53 (0.39–0.72); β-carotene, 0.49 (0.37–0.66); lutein with zeaxanthin, 0.59 (0.44–0.80); total lutein with zeaxanthin, 0.56 (0.41–0.76)) and model 2 (OR_Q4 vs. Q1_ (95% CI), α-carotene, 0.60 (0.43–0.83); β-carotene, 0.57 (0.41–0.80); lutein with zeaxanthin, 0.69 (0.51–0.94); total lutein with zeaxanthin, 0.66 (0.48–0.89)). In model 3, individuals in the highest quartile of dietary α-carotene intake exhibited a 31% reduction in odds of constipation when compared to those in the lowest quartile intake (OR_Q4 vs. Q1_ = 0.69, 95% CI: 0.48–0.98, p for trend = 0.04).

**Fig. 2:**
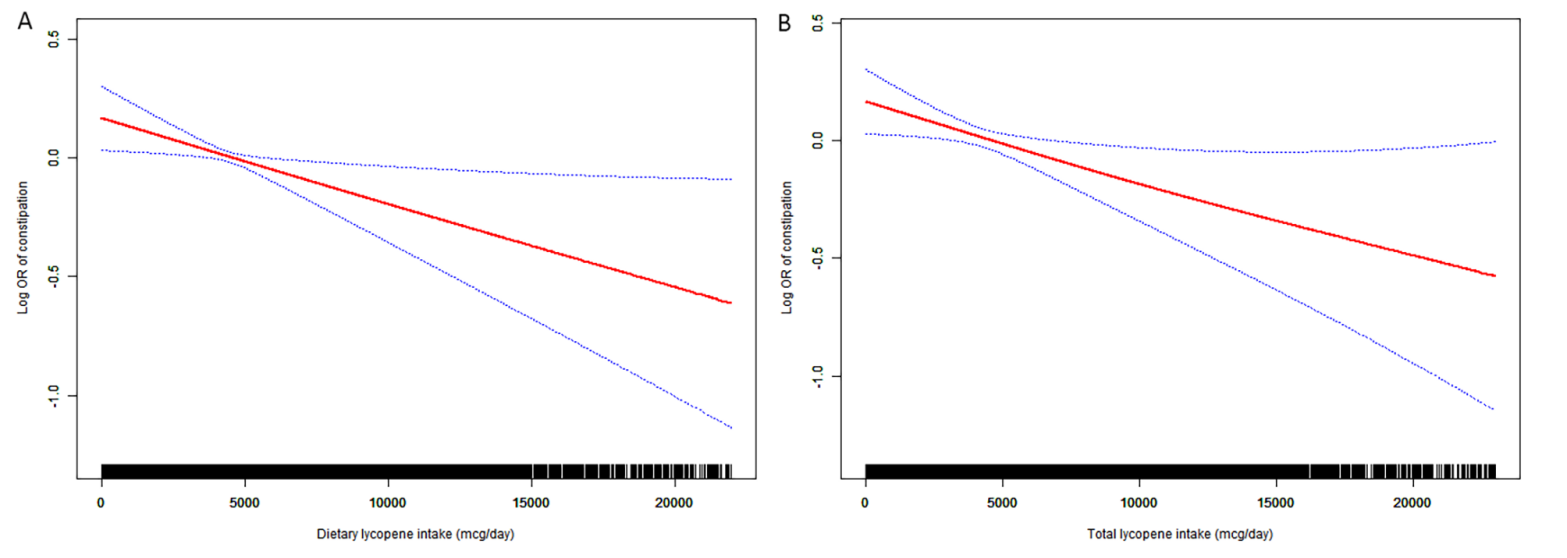
Weighted smooth curve fitting among men The weighted smooth curve fitting is used to display the association between dietary lycopene (2 A), total lycopene (2B) intake and log-transformed odds ratio of chronic constipation based on the generalized additive model (GAM) among men. Adjusting for all covariates, including age, race, marital status, educational levels and PIR, vigorous physical activity, BMI, smoking, drinking, diabetes, hypertension, depression, and dietary energy, dietary fiber, fat, fluid intake, and HEI 2015 scores. The red line represents the fitted line and the blue line refers to the 95% confidence intervals

**Fig. 3:**
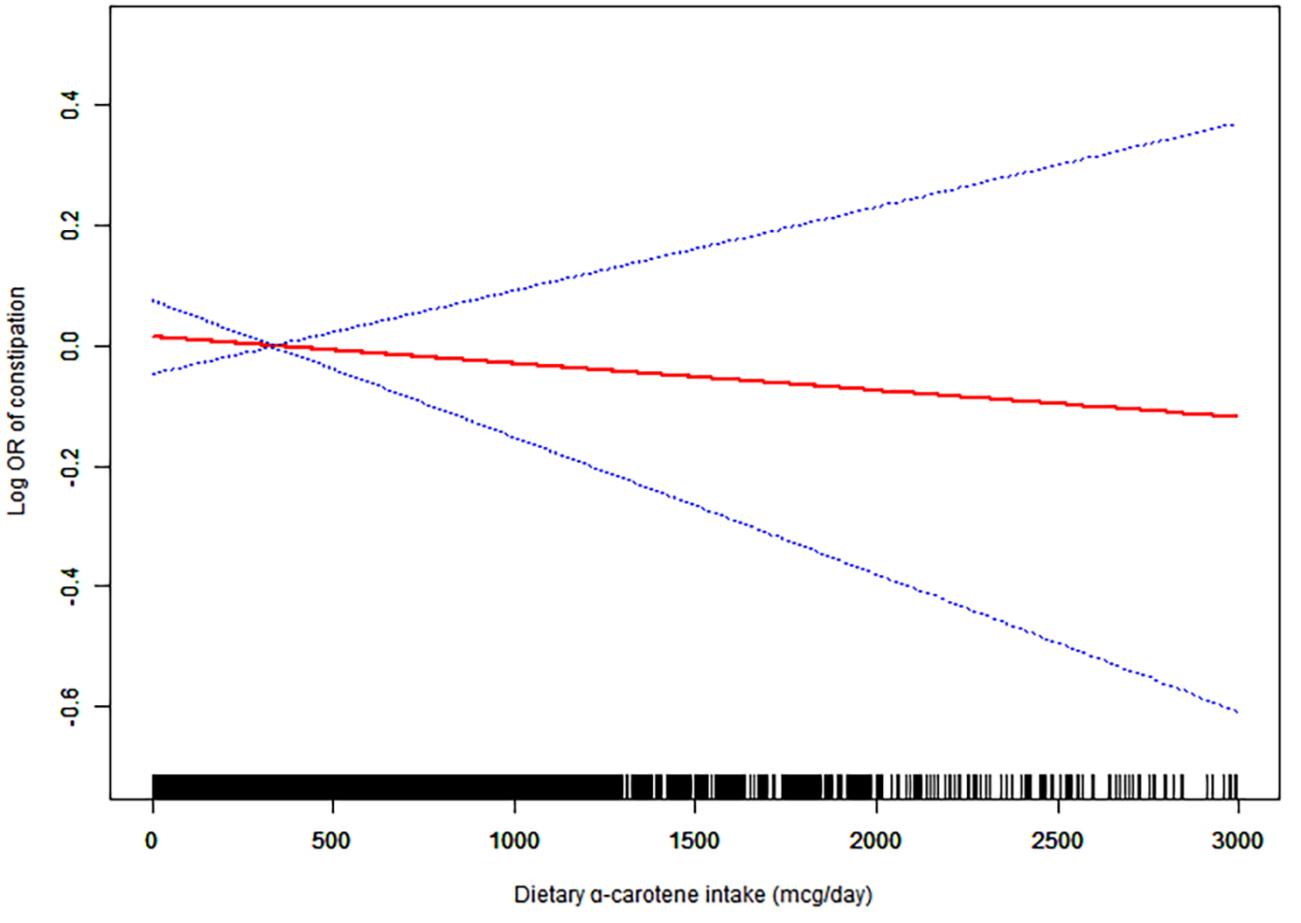
Weighted smooth curve fitting among women The weighted smooth curve fitting is used to display the association between dietary α-carotene and log-transformed odds ratio of chronic constipation based on the generalized additive model (GAM) among women. The covariates of age, race, marital status, educational levels and PIR, vigorous physical activity, BMI, smoking, drinking, diabetes, hypertension, depression, and dietary energy, dietary fiber, fat, fluid intake, and HEI 2015 scores were adjusted. The red line represents the fitted line and the blue line refers to the 95% confidence intervals

Weighted smoothed curve fitting based on the GAM further demonstrated linear dose-response relationships between dietary lycopene intake in men and dietary α-carotene intake in women and the risk of chronic constipation (Figs. 2 and 3).

### Results of subgroup analysis

**Fig. 4:**
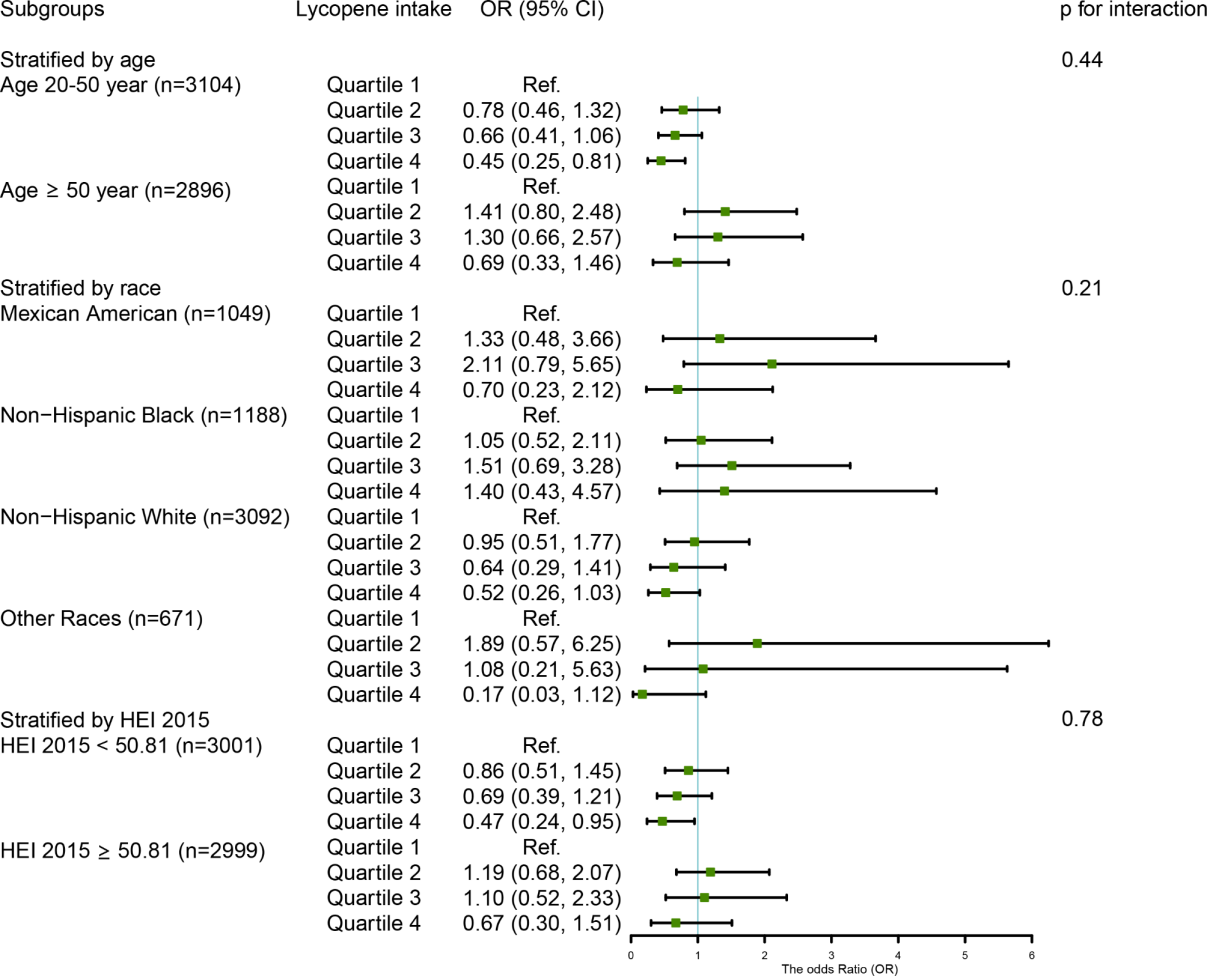
The weighted stratified and interaction analysis of relationship between dietary lycopene intake and covariates in men Age, race, marital status, educational levels, PIR, vigorous physical activity, BMI, smoking, drinking, diabetes, hypertension, depression, and dietary energy, dietary fiber, fat, fluid intake, and HEI 2015 scores were adjusted, except for the corresponding variables

**Fig. 5:**
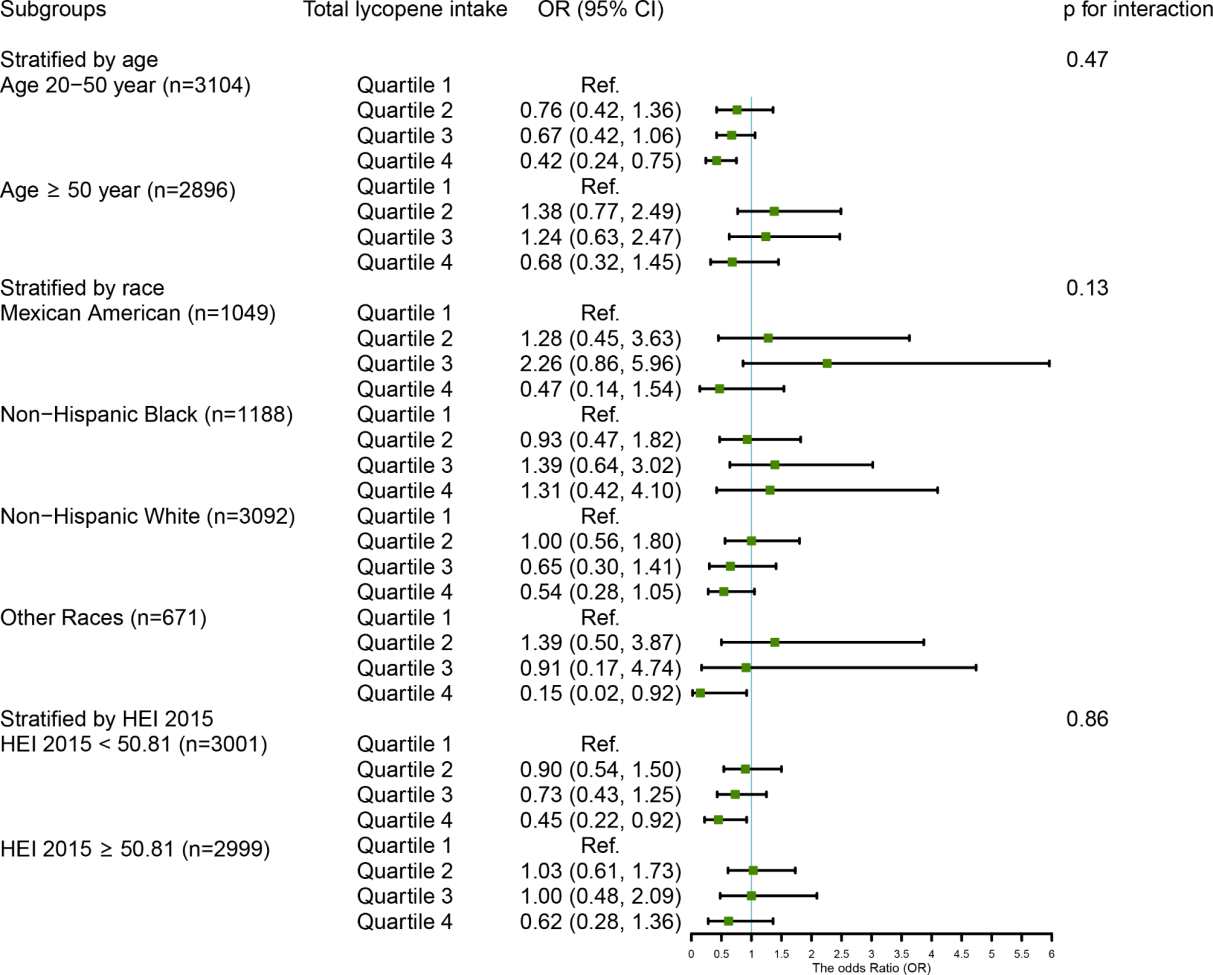
The weighted stratified and interaction analysis of association between total lycopene intake and covariates in men The covariates include age, race, marital status, educational levels, PIR, vigorous physical activity, BMI, smoking, drinking, diabetes, hypertension, depression, and dietary energy, dietary fiber, fat, fluid intake, and HEI 2015 scores, except for the corresponding variables

**Fig. 6:**
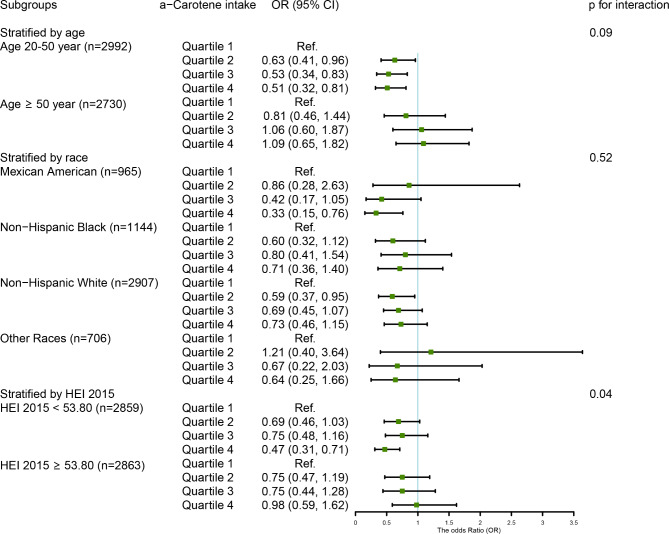
The relationship between α-carotene intake and covariates in women was assessed by weighted stratified and interaction analysis The covariates include age, race, marital status, educational levels, PIR, vigorous physical activity, BMI, smoking, drinking, diabetes, hypertension, depression, and dietary energy, dietary fiber, fat, fluid intake, and HEI 2015 scores, except for the corresponding variables

We observed that among men, the inverse association between the highest consumption of dietary lycopene and total lycopene and the risk of chronic constipation were particularly significant in participants of younger (OR_Q4 vs. Q1_ = 0.45, 95% CI: 0.25–0.81, p for trend = 0.01; OR_Q4 vs. Q1_ = 0.42, 95% CI: 0.24–0.75, p for trend = 0.01, respectively) and lower scores of HEI-2015 (OR_Q4 vs. Q1_ = 0.47, 95% CI: 0.24–0.95, p for trend = 0.04; OR_Q4 vs. Q1_ = 0.45, 95% CI: 0.22–0.92, p for trend = 0.04, respectively), as compared to those with the lowest consumption. No interaction was found between them (all p for interaction > 0.05) (Figs. 4 and 5). In women, the protective relationship between higher intake of α-carotene and constipation was robust in younger individuals (OR_Q4 vs. Q1_ = 0.51, 95% CI: 0.32–0.81, p for trend = 0.003), Mexican Americans (OR_Q4 vs. Q1_ = 0.33, 95% CI: 0.15–0.76, p for trend = 0.004), and those with an unhealthy diet (OR_Q4 vs. Q1_ = 0.47, 95% CI: 0.31–0.71, p for trend = 0.002). The interaction results indicated that the impact of α-carotene intake on chronic constipation varied according to HEI 2015 scores (p for interaction = 0.04) (Fig. 6).

## Discussion

To our knowledge, this is the first investigation of the association between the intake of carotenoids and chronic constipation in a large, nationally representative sample of adults. The overall weighted prevalence of constipation was 8.08%, with a significantly higher prevalence among females in comparison to males. Despite being slightly lower than findings reported in previous analyses, chronic constipation continues to pose a substantial global burden of disease. Dietary and lifestyle management is an initial strategy to prevent and treat chronic constipation, therefore, there is a pressing need to enhance research efforts in this area to bolster the management of the problem.

This study discovered a noteworthy inverse relationship between the intake of lycopene and chronic constipation among males (OR_Q4 vs. Q1_ = 0.55, 95% CI: 0.36–0.84 for dietary lycopene, OR_Q4 vs. Q1_ = 0.52, 95% CI: 0.34–0.80 for total lycopene), whereas the intake of α-carotene was negatively linked to constipation among females (OR_Q4 vs. Q1_ = 0.69, 95% CI: 0.48–0.98). Meanwhile, higher lycopene intake in men exhibited a more protective effect in younger (OR_Q4 vs. Q1_ = 0.45, 95% CI: 0.25–0.81 for dietary lycopene; OR_Q4 vs. Q1_ = 0.42, 95% CI: 0.24–0.75 for total lycopene) and lower HEI 2015 scores individuals (OR_Q4 vs. Q1_ = 0.47, 95% CI: 0.24–0.95 for dietary lycopene; OR_Q4 vs. Q1_ = 0.45, 95% CI: 0.22–0.92 for total lycopene), while the advantageous impact of α-carotene intake was more pronounced in younger women (OR_Q4 vs. Q1_ = 0.51, 95% CI: 0.32–0.81), Mexican Americans (OR_Q4 vs. Q1_ = 0.33, 95% CI: 0.15–0.76) and those with lower HEI 2015 scores (OR_Q4 vs. Q1_ = 0.47, 95% CI: 0.31–0.71). There was an interaction between HEI 2015 scores subgroup and α-carotene intake against constipation. This was of great interest because the management of constipation is still a serious challenge, and increasing certain carotenoids intake through dietary changes may be an effective way to prevent and treat it, especially in particular subgroups.

Age and gender are important risk factors for the occurrence of constipation [17, 18]. In our study, the prevalence of constipation was found to be significantly higher among women than men (more than twice as high), but we did not observe an increase in prevalence with age, and these results could be supported by previous findings from NHANES [8]. Furthermore, we observed that the protective effect of lycopene was more significant among younger men, potentially attributed to its significantly impaired bioavailability in older individuals [19]. Liu et al. [20] noted that a higher overall dietary quality has the potential to mitigate the risk of chronic constipation, but in our results, the protective impact of lycopene among men and α-carotene among women against constipation was more pronounced in individuals with lower HEI 2015 scores, suggesting that higher carotenoids intake may be affiliated with specific dietary components regardless of overall quality of the diet. Further research is necessary to validate these findings.

Lycopene, which is mainly found in tomatoes and its products [21], has powerful anti-inflammatory and free radical scavenging effects [22, 23], and its implications for intestinal function are being intensively studied. Several possible reasons could explain the protective association between dietary lycopene intake and chronic constipation. First, it could alleviate intestinal inflammation and inhibit oxidative stress. Animal studies have suggested that lycopene could not only suppress the expression of pro-inflammatory factors like IL-1β, IL-6, IL-8 and TNF-α, but also stimulate the production of anti-inflammatory cytokines (such as TGF-β, IL-10), so as to reduce intestinal inflammation [24, 25]. Under conditions of oxidative stress, the system exhibited an overproduction of reactive oxygen species (ROS), resulting in detrimental effects on the intestinal mucosa and subsequent impairment of intestinal function. Lycopene effectively reduced the adverse effects of drugs and radiation on oxidative stress markers such as myeloperoxidase (MPO), superoxide dismutase (SOD), catalase (CAT), glutathione (GSH), glutathione peroxidase (GSH-P_x_) and malondialdehyde (MDA) in intestine, thereby maintaining intestinal health [24, 26, 27].

Secondly, lycopene has the potential to uphold normal intestinal barrier function. The barrier is comprised of four components, namely the mechanical, immune, chemical, and microbial barrier. Several tight junction proteins are involved in forming the intestinal mechanical barrier, and they could regulate intestinal permeability by connecting the cellular gaps. Wang et al. [24] suggested that lycopene significantly inhibited the disruption of tight junction proteins induced by sulfamethoxazole (SMZ) by increasing the transcriptional levels of Claudin1, Claudin4, Occludin and ZO-1, indicating that lycopene could regulate the expression of tight junction proteins to maintain the intestinal epithelial barrier; similar results were found in a series of animals studies [25, 26, 28]. Moreover, secretory IgA (sIgA) serves as the first defenses of intestinal mucosal immunity, effectively binding to pathogens and preventing them from damaging the intestine. IL-4, IL-12 and IFN-γ are important immunomodulatory cytokines that could reflect the status of both humoral and cellular immunity [29]. In an animal model of intestinal ischemia-reperfusion, it has been documented that the preoperative administration of lycopene limited the loss of intestinal IgA and sIgA levels, as well as decreased bacterial translocation [30]. Pan et al. [31] observed that lycopene pretreatment reduced the decline in intestinal mucosal sIgA levels induced by cyclophosphamide and stimulated the secretion of IL-4, IL-12 and IFN-γ by activating the TLR4-MyD88/TRIF-TRAF6 signaling pathway. These results imply that lycopene could potentially recover the normal immune function of the mucosa.

Thirdly, lycopene could regulate intestinal bacteria. For example, Bifidobacterium and Lactobacillus, which are widely recognized probiotics, exhibit substantial advantages in the treatment of gastrointestinal and systemic disorders [32, 33]. Lycopene could alter the composition of gut microbiome in dextran sodium sulfate (DSS) treated colitis mice by enhancing the relative abundances of Bifidobacterium and Lactobacillus and decreasing the relative abundances of the Proteobacteria [28]. In vitro Fermentation Model, an increasing abundance of Bacteroidetes and Proteobacteria was observed following lycopene treatment in comparison to the blank control group [34]. In addition, bacterial metabolites could have an impact on intestinal function. Short-chain fatty acids (SCFAs) are important metabolites produced by intestinal microorganisms that play a crucial role in preserving normal colon function, as well as maintaining the typical morphology and features of colonic epithelial cells. Zhao et al. [28] showed that lycopene could increase the levels of SCFAs such as acetate, iso butyric acid, valeric acid, and isovaleric acid in colitis mice. These results support a positive role for carotenoids in intestinal health.

Consequently, lycopene may promote intestinal health and ultimately ameliorate chronic constipation by rectifying intestinal microbial disorders, mitigating inflammation and oxidative stress, and maintaining normal intestinal mucosal epithelial barrier function.

α-Carotene (pro-vitamin A) is more abundant in carrots and pumpkins [32]. Similar to lycopene, it also has anti-inflammatory and antioxidant properties, but its influence on intestinal function has received limited investigation. A multi-ethnic cohort study reported that participants consuming carotenoids-rich fruits and vegetables may exhibit a higher alpha diversity of intestinal flora [36]. Similarly, the levels of dietary and plasma concentrations of α-carotene in pregnant women were positively associated with the alpha diversity of their fecal microbiota. Furthermore, the beta diversity of the gut microbiota displayed significant variations in relation to reported consumption of carotenoid-containing foods and the plasma levels of α-carotene. However, these findings may be driven by enhanced overall diet quality or elevated fiber intake, rather than a specific impact of carotenoids [37]. Therefore, further examinations regarding its impacts and potential mechanisms on gut function are warranted.

In this study, we observed that the protective effects of lycopene and α-carotene on constipation exhibit gender differences. Hormonal and metabolic variations between men and women may contribute to these disparities. First, Women’s hormonal fluctuations throughout the menstrual cycle can impact gastrointestinal motility and transit time [38], which may mask or diminish the effects of lycopene on constipation, while hormones like estrogen may interact with α-carotene [39, 40], leading to a distinct association. Second, men and women exhibit differences in the absorption, metabolism, and utilization of carotenoids [41], which may influence their effects on gastrointestinal health. We did not observe the associations between constipation and other carotenoids in this study which could be attributed to several factors, including differences in their structure, environmental polarity, absorption, metabolism, bioavailability, and interactions with gastrointestinal health [41–43].

There are several strengths of this research. Primarily, it is the first study to assess the association between dietary carotenoids intake and constipation among both genders, effectively addressing a void in existing research in this field. Furthermore, we utilized large sample data from NHANES with proper sample weights, which can be representative of the non-institutional population of the United States. Additionally, we conducted adjustments for potential confounding variables that would be linked to carotenoids and constipation, thereby enhancing the credibility of our study findings. Consequently, our research outcomes hold promise in offering novel insights into dietary strategies for managing constipation.

The study also has some limitations. First, NHANES collected cross-sectional data, so the causal relationship between increasing dietary carotenoids intake and constipation cannot be determined. Second, recall bias is unavoidable because dietary data are acquired from 2-day dietary questionnaires. Third, we only calculated the carotenoids in dietary and supplements, without taking into account their bioavailability. Fourth, the potential influence of medication usage, such as antidepressants, on the occurrence of constipation was not accounted for due to limited access to complete data. As a consequence, it is necessary to conduct more basic and clinical studies in the future to confirm what we found and investigate the mechanisms involved.

## Conclusions

Overall, we found that dietary lycopene intake was a protective factor against constipation among men, whereas α-carotene intake was negatively associated with the prevalence of constipation among women. These results may offer people the opportunity to better manage their disease by changing their diet.

## Data Availability

The datasets generated and/or analyzed during the current study are available in the NHANES database, https://wwwn.cdc.gov/nchs/nhanes/.

## Abbreviations

NHANES: National Health and Nutrition Examination Survey
BSFS: Bristol stool form scale
OR: Odds ratio
CI: Confidence interval
BHQ: Bowel health questionnaires
MEC: Mobile Examination Center
BMI: Body mass index
PIR: Poverty-income ratio
HEI: Healthy eating index
SE: Standard errors
GAM: Generalized additive model
